# Does the psychoemotional well-being of Spanish students influence their mathematical literacy? An evidence from PISA 2018

**DOI:** 10.3389/fpsyg.2023.1196529

**Published:** 2023-06-12

**Authors:** David Molina-Muñoz, José Miguel Contreras-García, Elena Molina-Portillo

**Affiliations:** Department of Didactics of Mathematics, University of Granada, Granada, Spain

**Keywords:** psychoemotional status, PISA, assessment, multilevel analysis, mathematical literacy

## Abstract

Data from international studies reveal that the mathematics literacy of Spanish students is significantly lower than that of students from nearby countries. Therefore, in recent years, interest in identifying the factors that influence students’ mathematics results in Spain has grown considerably. Often, these factors are sought among the socioeconomic characteristics of the students or among variables related to the schools, ignoring the psychological and emotional factors of the students. This paper analyzes the impact of certain psychoemotional characteristics of Spanish students on their literacy in mathematics. For this purpose, multilevel regression models are applied to the data of the Spanish sample of the 2018 edition of PISA (Programme for International Student Assessment), which is composed of 35,943 15-year-old students. The instruments for data collection are the mathematics literacy tests and the contextual questionnaires on students’ personal situation and well-being used by PISA. As dependent variable, students’ mathematics literacy has been considered, measured through the plausible values provided by PISA, and as independent variables, different indices measuring students’ psychoemotional well-being obtained from the contextual information collected by PISA. Results indicate that resilience, motivation for the achievement of learning objectives, competitiveness, perceived cooperation at school, and social connectedness with parents have a positive impact on students’ mathematics literacy, while experiences related to bullying, physical self-concept, meaning in life and perceived competitiveness at school have a negative impact.

## 1. Introduction

It is widely found that the subject of Mathematics often stands out as being one of the subjects in which students tend to most under-perform (Gamboa Araya, 2014). In Spain, this assertion in supported by the findings of studies with international reach, such as those of the TIMSS (Trends in Mathematics and Science Study) and the PISA (Programme for International Student Assessment). Indeed, Mathematics results for Spanish students are in line with data produced by these studies and are found to be lower than those obtained by students from the majority of surrounding countries (Ministry of Education and Professional Training, 2019, 2020). Consequently, in recent years, interest in better understanding the potential factors to influence Mathematics literacy in Spanish students has grown significantly within the educational community with the aim of addressing this issue. In an attempt to identify these factors, studies have mainly focused on cognitive variables in relation to students (González-Pienda, 2003). This is due, in part, to the tendency to hold cognitive aspects in higher regard than all other aspects when it comes to educational processes (Fernández-Berrocal and Ruiz, 2008; García-Retana, 2012). Nonetheless, since the end of the last century, work such as that conducted by Salovey and Mayer (1990), Gardner (1995), Goleman (1996), and Bar-On (1997) began to highlight the importance of emotions in learning. Thanks to this, consideration of the emotional dimension has become more and more importance in educational processes. Such is this the case that some authors have urged the need to broaden the traditional understanding of intelligence to make room for psychological and emotional qualities, which differ from and, yet, complement cognitive capacities (Dueñas-Buey, 2002). In fact, it has even been suggested that students acquire certain emotional skills prior to gaining access to traditional academic material (Jiménez and López-Zafra, 2009).

The effects of the psychological and emotional state of students on their academic outcomes has been broadly documented in existing literature on different educational stages (Extremera-Pacheco and Fernández-Berrocal, 2004; Robles-Ojeda et al., 2011; Ferragut and Fierro, 2012; Ros-Morente et al., 2017). In general terms, the majority of conducted studies have highlighted that negative emotional states can have unfavourable repercussions on student learning processes and, consequently, their academic results, whilst the presence of good emotional well-being promotes efficient cognitive processes (Elizondo-Moreno et al., 2018).

The complexity of a student’s psychoemotional status makes it difficult to study it directly. For this reason, the concept has been approached in a number of ways based on determined indicators or components that are easier to address. The components of a student’s psychoemotional health can be classified as personal or relational. According to Perpiñán (2013), the former pertains to components that are related with understanding and control inherent to the student themselves, whilst the latter refer to the relationship and interaction of the student with individuals. Existing scientific literature does not reveal any clear consensus regarding the influence of individual components, such as aspects including perceptions of physical appearance, resilience, assertiveness, perseverance, self-esteem or stress management, of academic outcomes. Indeed, of the individual variables analysed by Broc (2019), only the management of stress was found to make a significant contribution towards explaining academic results in a group of secondary school students. Along the same lines, whilst Ruíz-Melero et al. (2020) reported evidence that stress management significantly correlated with student achievement in subjects from four different ambits (linguistic, scientific, social and artistic), other individual skills, such as self-esteem and assertiveness, only correlated with results in subjects that were artistic in nature but not in all other examined ambits. In contrast to these findings, a study conducted by Buenrostro-Guerrero et al. (2012) concluded that both stress management in students and their personal emotional abilities were found to be significantly related with performance at school. With regards to the relational component of psychoemotional health, it can be inferred that the emotional state of students is not only influenced by them themselves but is also conditioned by agents outside of their social setting. Of all of these agents, family, in general, and parents, in particular, play a central role in the emotional development of students. Family makes up the first social network of all individuals and constitutes a context charged with emotions that undoubtedly influences emotional development, especially during the stages of childhood and adolescence, and has repercussions in all ambits of life (Pichardo-Martínez et al., 2002; Orcasita-Pineda and Uribe-Rodríguez, 2010; García-Navarro, 2011; Márquez Cervantes and Gaeta González, 2017). With regards to the academic setting, parental involvement in their children’s school development fosters positive attitudes and perceptions towards school in their children, which lead to improved academic performance of students (Jungert et al., 2020). In this line, authors such as Sánchez and Dávila (2022) and Lastre Meza et al. (2018) have highlighted that solid emotional accompaniment of parents to their children promotes their child’s academic success. Finally, another aspect that influences an individual’s emotional well-being refers to the experiences at the school. The school is a place not only for academic training, but also for personal and social development where a student spends a large part of the day (Kleinkorres et al., 2023). Authors such as Ryan and Deci (2020) point out that aspects such as confidence, self-esteem and mental health are profoundly affected by school students’ experiences in their school. As a consequence, the concept of school well-being has recently emerged and has been studied by several authors, most of whom agree in including aspects related to students’ affect and emotions derived from their experiences at school when defining it (Aulia et al., 2020). These experiences are strongly conditioned by the climate perceived to be present in the classroom and at the educational institution. In fact, the school climate has been found to be directly related with academic performance in students, with this relationship sometimes being mediated by the emotional competence of students (Espinoza, 2006; Wang and Holcombe, 2010; López-González and Oriol, 2016). In the same way, the experience of violence or abuse in the school setting, as with any form of abuse, leads to a decline in emotional health and, with it, a reduction in school grades for both victims and aggressors (Garaigordobil, 2011; Martínez-Rodríguez, 2017).

In Mathematics, authors such as McLeod (1989, 1992), Gil et al. (2005), and Blanco et al. (2010) have highlighted the importance of emotions in the teaching-learning processes pertaining to the subject. In work conducted in 1992, McLeod proposed that the emotional dimension in the teaching of Mathematics should be addressed by considering three main components: beliefs, attitudes and emotions. Since then, a number of authors have examined the relationship that exists between these components and results in Mathematics in students at different educational stages. The majority of these authors have concluded that significant positive relationships exist between emotions and performance (Mato and de la Torre, 2010; Molera, 2012; Muñoz-Cantero et al., 2018). McLeod’s approach to the affective dimension is exclusively designed for the area of mathematics in the sense that the beliefs, attitudes and emotions that make is up describe affective responses to situations related with the subject of Mathematics (McLeod, 1992). Nonetheless, for some time now, examinations of the affective dimension in Mathematics have been conducted alongside analyses of other abilities that are not only related to this subject but, instead, are more holistic in nature and reflect the psychoemotional status of individuals. This has given rise to the concept of the emotionally literate individual in Mathematics. This covers everything involved in the development of an individual’s emotional intelligence in the context of Mathematics and the way they manage to find a way to interact with this ambit, whilst also taking into account both one’s own feelings and emotions as well as those of others (Gómez-Chacón, 2000). Consequently, research has begun to examine the influence of student’s general psychoemotional state on Mathematics achievement. Gallesi and Matalinares Calvet (2012) found a significant and positive correlation between logic-mathematics performance of a group of primary school students and their resilience, understood as a global response that activates protective mechanisms and enables the individual to come away strengthened from a situation that is, at first, perceived to be adverse (Rutter, 1991). Specifically, of the five dimensions considered by this study and used to measure resilience in students, three (self-esteem, empathy and autonomy) were found to be significantly related with performance. Similarly, according to the study by Donolato et al. (2020), resilience was also found to have a significant effect on the mathematics performance of a group of Italian middle school students. In the same line, Ros-Morente et al. (2017) reported that Mathematics performance in a group of students could be predicted, to a large degree, from their emotional develop and self-esteem. In a study conducted with a group of secondary school students, Salcedo-Rodríguez and Pérez-Vázquez (2020) demonstrated that motivation and empathy was weakly, yet significantly, associated with the mathematic ability.

The importance of emotions as an indicator of the global health of students and their impact on academic performance has led educational studies with international reach, such as the aforementioned PISA, to start to gather information on the individual well-being of participants. PISA is an international macro study ran by the Organisation for Economic Co-operation and Development with the aim of identifying the skills held by 15-year-olds in relation to the three areas of mathematics, science and reading comprehension. To this end, participating students performed a series of tests in order to demonstrate their skills and abilities for each of the three ambits mentioned above. In addition, PISA gathers a huge amount of information about the context in which students operate. Such information includes variables pertaining to the family situation, school situation and, in addition, variables that are individual or personal in nature. In the 2015 version of the PISA, data was collected for the first time on the well-being of participating students as a part of the information gathered on their personal state. This made it the first international and educational macro study of its kind (OECD, 2019a). Indeed, in the PISA 2015 questionnaire on students’ personal and family background, some questions were included to collect students’ perceptions of their well-being. Given the limitations in the scope of the conclusions that could be drawn from these questions, it was decided in the 2018 edition to create a separate questionnaire to collect information on all dimensions of participants’ well-being (OECD, 2019a). According to the PISA, student well-being is structured according to three different dimensions. The first is an individual dimension and concerns health status and perceptions about the way in which students feel about themselves and their lives. The second is an academic dimensions and concerns student perceptions about their day-to-day at the institution in which they are undertaking their studies. Finally, the social dimension concerns student perceptions about the relationships they form outside of the educational institution with their parents, their friends, etc. Further, this dimension encapsulates student perceptions about their life in a general sense (OECD, 2019a). The inclusion of this type of information within the data provided by the PISA has led some authors to start to examine relationships between factors that are psychoemotional in nature and studentliteracy. Specifically, Antonelli-Ponti et al. (2021) demonstrated that both the emotional support provided by parents and the sense of belonging at school positively correlated with literacy in the three areas evaluated by PISA 2015 in Brazilian students. In the same study, the sense of belonging at school was also found to have a significant impact on the literacy in these three examined areas. Another variable in the school environment that significantly influences student literacy according to studies based on the analysis of PISA data is the classroom climate (Ruíz de Miguel, 2009; de Jorge-Moreno, 2016). In Spain, the work of Gil-Madrona et al. (2019), based on the analysis of PISA 2015 data, found that while students’ anxiety had a negative impact on their science literacy, their sense of belonging to the school and cooperation among students had a positive impact. On the other hand, Rusteholz and Mediavilla (2022) showed bullying to have a negative impact on the literacy in all of the areas evaluated by the PISA in 2018. In real terms, this impact was found to equivalate to the loss of 3–5 months of formal education for victimised students when compared with their non-victimised peers. Nevertheless, very few studies have examined the effect of variables that are psychoemotional in nature on mathematic literacy in Spanish students.

Based on that discussed above, the aim of the present work was to identify the psychoemotional variables that impact the mathematical literacy of Spanish students and quantify their effect by analysing data provided by the PISA in 2018.

## 2. Materials and methods

The sample used in the present study corresponds to the Spanish sample of the 2018 edition of the PISA. Specifically, the sample is composed of 35,943 students aged 15 years, of which 50.05% are male and the remainder are female.

The dependent variable specified when conducting modelling analysis was student literacy in the subject of Mathematics, which is defined as an individual’s capacity to formulate, employ, and interpret mathematics in a variety of contexts (OECD, 2019d). The PISA measured mathematical literacy according to measures known as plausible values. In the PISA, plausible values are representations of the range of potential scores used to evaluate students. The values are drawn randomly from the probability distribution pertaining to each student’s marks or grades, the calculation of which considers the responses given by students to the mathematical tests they complete, in addition to some variables pertaining to context (OECD, 2019b). Plausible values are scaled in such a way that, approximately, they produce a mean of 500 and a standard deviation of 100, when drawn from all of the countries that make up the OECD. In 2018, the PISA produced 10 plausible values for Mathematics. Thus, each one of these 10 values will be examined as an independent variable and the outcomes of these analyses will be combined in line with that outlined by the OECD (2009).

As independent variables, the present study considered the indices provided by the PISA that reflect the psychoemotional state of students. These indices are presented in Table 1.

**Table 1:** Examined independent variables.

Ambit	Variable	Description
Individual	COMPETE	Competitiveness
	FEARFAIL	Fear of failure
	EUDMONIA	Perception of purpose or meaning in life
	POSFEEL	Perception of positive feelings (happiness, joy…)
	RESILIENCE	Resilience
	BODYIMAGE	Satisfaction with appearance and body image	WORKMAST	Motivation to improve and task mastery through effort	MASTGOAL	Motivation to achieve goals and maximise learning
School	BELONGSCH	Sense of belonging at the academic institution
	BULLYING	Experiences of bullying suffered by the student
	PERCOOP	Perception of cooperation by students at the academic institution
	PERCOMP	Perception of competitiveness by students at the academic institution
Social	PAREMOSUP	Perceived emotional support received from parents	SOCONPAR	Feelings of social connection and attachment to parents

The PISA estimates values for these indices for all students as a function of the responses they provide to a series of questions contained in the questionnaire on their personal and family context and in the questionnaire on their well-being. Table 2 shows the items used for the calculation of each of the indices.

**Table 2:** Items considered by PISA for the calculation of the independent variables.

Variable	Items
COMPETE	I enjoy working in situations involving competition with others.
	It is important for me to perform better than other people on a task.
	I try harder when I’m in competition with other people.
FEARFAIL	When I am failing, I worry about what others think of me.
	When I am failing, I am afraid that I might not have enough talent.
	When I am failing, this makes me doubt my plans for the future.
EUDMONIA	My life has clear meaning or purpose.
	I have discovered a satisfactory meaning in life.
	I have a clear sense of what gives meaning to my life.
POSFEEL	Thinking about yourself and how you normally feel: how often do you feel joyful?
	Thinking about yourself and how you normally feel: how often do you feel cheerful?
	Thinking about yourself and how you normally feel: how often do you feel happy?
RESILIENCE	I usually manage one way or another.
	I feel proud that I have accomplished things.
	I feel that I can handle many things at a time.
	My belief in myself gets me through hard times.
	When I’m in a difficult situation, I can usually find my way out of it.
BODYIMAGE	I like my look just the way it is.
	I consider myself to be attractive.
	I like my body.
	I like the way my clothes fit me.
WORKMAST	I find satisfaction in working as hard as I can.
	Once I start a task, I persist until it is finished.
	Part of the enjoyment I get from doing things is when I improve on my past performance.
MASTGOAL	My goal is to learn as much as possible.
	My goal is to completely master the material presented in my classes.
	My goal is to understand the content of my classes as thoroughly as possible.
BELONGSCH	I feel like an outsider (or left out of things) at school.
	I make friends easily at school.
	I feel like I belong at school.
	I feel awkward and out of place in my school.
	Other students seem to like me.
	I feel lonely at school.
BULLYING	Other students left me out of things on purpose.
	Other students made fun of me.
	I was threatened by other students.
PERCOOP	Students seem to value cooperation.
	It seems that students are cooperating with each other.
	Students seem to share the feeling that cooperating with each other is important.
PERCOMP	Students seem to value competition.
	It seems that students are competing with each other.
	Students seem to share the feeling that competing with each other is important.
PAREMOSUP	My parents support my educational efforts and achievements.
	My parents support me when I am facing difficulties at school.
	My parents encourage me to be confident.
SOCONPAR	My parents help me as much as I need.
	My parents show that they care.
	My parents try to understand my problems and worries.
	My parents encourage me to make my own decisions.

OECD (2019c).

Each of the items is answered by students using a Likert scale with four or five response categories, depending on the item. The responses to the items constituting each index are then modelled and subsequently scaled to produce the index itself, which is continuous in nature. The scaling process is carried out in such a way that the mean of each index is equal to 0 when all students from OECD countries are considered. Positive variable ratings suggest more positive responses in relation to the average student from OECD countries, whilst negative ratings suggest more negative responses than these students (OECD, 2019b). At the time of analysing information provided by the PISA, it is necessary to consider the hierarchical nature of the data obtained. This hierarchy responds to the fact that each individual student is enrolled at a particular school or educational institution. As a result, characteristics inherent to the institution can exert a certain degree of influence over student literacy, meaning that the assumption of independence does not apply to the observations made. This means it is not possible to apply traditional techniques of linear regression, given that the fulfilment of this assumption is an essential requisite. Instead, hierarchical regression techniques, also known as multilevel regression (Hox, 2002; Cebolla-Boado, 2013) consider the dependence between observations at the time of modelling outcomes, making them much more appropriate for the analysis of data produced by the PISA. Multilevel regression breaks down the total variability of observations as a function of the nested levels included within the model and indicates the proportion of variance that can be attributed to each level. Data analysis in the present work considered two levels. Firstly, the student level and, secondly, the school level. Modelling normally starts by estimating known parameters and producing a null model, which does not consider any predictor when producing an estimation. Following this, the independent variables from one of the two levels or from both of the levels can be included in the model in order to explain variability in the response variable. In the present study, the independent variables considered described student characteristics, meaning that all independent variables pertained to the student level.

Data were analysed using version 25 of the statistical analysis software SPSS (IBM Corporation, 2017). Estimations were considered to be significant when the absolute value of estimation was 2 times greater than its corresponding standard error term.

## 3. Results

Firstly, this section will present outcomes of preliminary descriptive analysis conducted of the dependent variable and the independent variables. Next, the estimations produced by the multilevel regression model will be presented.

According to the outcomes presented in Table 3, literacy by Spanish students in the subject of Mathematics ranges between scores of 127.46 and 808.39, pertaining to a range of 680.93. Despite this range, scores pertaining to the middle 50% of students ranged between 432.39 and 553.25. The average score indicating the literacy of participating students in Mathematics was 491.16, placing them slightly below the average literacy of students from the OECD, which pertains to a score around 500. In the same way, the standard deviation pertaining to literacy in Mathematics was 87.54, which is lower than the average standard deviation of around 100 reported for OECD countries.

**Table 3:** Descriptive statistics for the dependent variable.

	Minimum	Mean	25^th^ perc.	Median	75^th^ perc.	Standard deviation	Maximum
Mathematical literacy	127.46	491.16	432.39	495.45	553.25	87.54	808.39

perc. = percentile.

**Figure 1:**
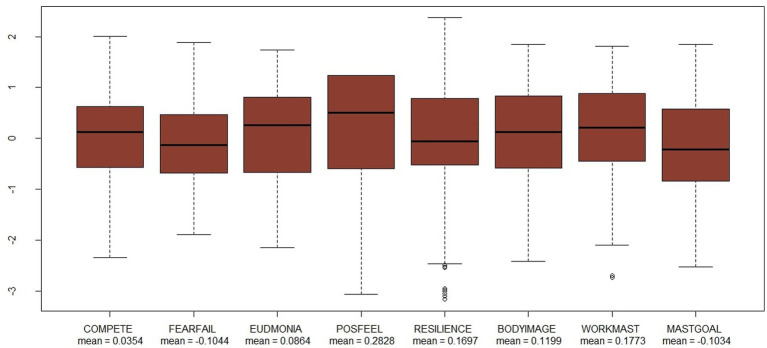
Box-and-whisker plots for the personal indices independent variables.

**Figure 2:**
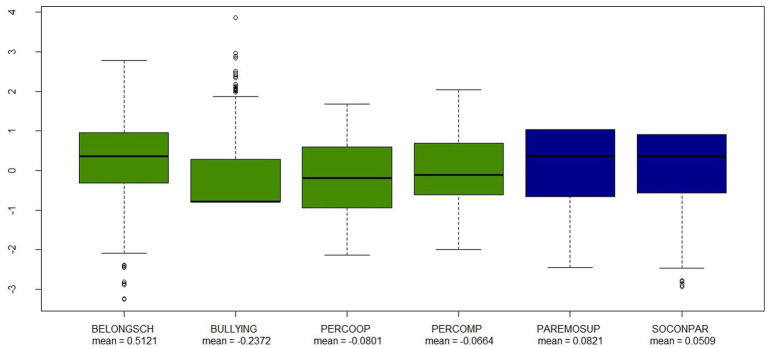
Box-and-whisker plots for the school (in green) and social (in blue) indices independent variables.

Next, Figures 1, 2 presents box and whisker plots of data pertaining to the independent variables considered in the regression model.

According to data presented in the figure presented above, Spanish students reported high levels of competitiveness (0.0354), with this level being slightly higher than the average reported for students from OECD countries (with this being 0). When compared with the average OECD student, the majority of students in the present sample had more clear perceptions of the meaning or purpose of their lives (0.0864). A noteworthy amount of variability was presented in relation to the index measuring perceived resilience in students. On the one hand, more than half of participating Spanish students gave negative indices of resilience. This suggests that, on average, the majority of these students considered themselves to be less resilient than OECD students. Indeed, some students reported extremely low levels of resilience when compared with the main bulk of the present sample. However, on the other hand, the average value for this index was positive, given that a group of students was found to report levels of resilience that were much higher than 0.

As shown in Figure 2, the majority of participating students in the present study felt a greater sense of belonging at school than the average student from OECD countries. Further, it was observed that Spanish students were less exposed to bullying at their educational institution than were students, on average, from OECD countries. Despite this, a reasonable number of students presented very high scores in relation to the bullying index.

Next, estimations produced by multilevel modelling analysis are presented, starting with those provided by the null model. These outcomes are presented in Table 4.

**Table 4:** Estimates and standard errors of the parameters of the null multilevel regression model.

Variables	Estimate	Standard error
Intercept	489.18	1.41
School level variance	1162.25	101.01
Student level variance	6563.93	115.46

The null model reveals that average literacy in Mathematics pertaining to Spanish students corresponds to a score of 489.18. Estimations pertaining to the variability existing both between schools and between students at each school were found to differ significantly from 0, with variability pertaining to the latter being much larger than that pertaining to the former. The deviance value produced for the null model was 419970.28.

For the next step in the modelling process, predictors were added to the model pertaining to the student level presented in Table 1. Estimations for this new model can be consulted in Table 5.

**Table 5:** Estimates and standard errors of the parameters of the multilevel regression model including student-level predictors.

Variables	Estimate	Standard error
Intercept	500.34	1.79
COMPETE	6.04	1.21
FEARFAIL	−1.59	1.51
EUDMONIA	−13.74	1.08
POSFEEL	−2.42	1.13
RESILIENCE	10.47	1.28
BODYIMAGE	−4.88	1.16
WORKMAST	−0.67	1.33
MASTGOAL	10.00	1.20
BELONGSCH	−0.87	1.08
BULLYING	−7.93	1.19
PERCOMP	−3.00	1.19
PERCOOP	5.04	1.23
PAREMOSUP	0.76	1.36
SOCONPAR	5.44	1.53
School level variance	764.67	55.62
Student level variance	5884.69	100.40

The multilevel model reveals that average literacy of Spanish subjects in the subject of Mathematics corresponded to a score of more than 500 when the value of all indices serving as independent variables was held at 0.

With regards to independent variables under examination pertaining to the individual component of psychoemotional well-being, variables describing resilience (RESILIENCE), motivation to achieve goals as fully as possible (MASTGOAL) and perceiving to have a clear meaning to life (EUDMONIA) had the greatest impact on mathematics literacy in students. The first two of these variables were positively correlated, indicating that, on the one hand, more resilient students and those showing greater interest in achieving greater mastery over academic content performed better in Mathematics. In contrast, the coefficient associated with the variable EUDMONIA was negative, with students reporting having a clearer purpose in life also tending to perform more poorly in Mathematics. Specifically, each point increase in indices measuring resilience, motivation to achieve goals and life purpose in students led to an increase of 10.47 and 10, and a decrease of 13.74, respectively, in their literacy on the subject when all other predictors were held stable. On the other hand, student competitiveness (COMPET) was directly related with their Mathematics score, in such a way that each unit increase in the index measuring this characteristic led to an increase of 6.04 in the score achieved by students in the subject of Mathematics. It can also be seen, that as student perceptions of their appearance and body image (BODYIMAGE) improved, their Mathematics score decreased. According to the developed model, neither fear of failing (FEARFAIL) nor the level of motivation to achieve academic improvement (WORKMAST) reported by students was associated with outcomes in Mathematics.

With regards to the indices used to indicate aspects of the psychoemotional state of students as a function of their relationship with their school environment, the index measuring exposure to bullying at school was shown to have the greatest impact when it came to explaining variability in Mathematics literacy. Students reporting having suffered *bullying* (BULLYING) at school were found to have significantly lower Mathematics scores, with scores being 7.93 points lower for each unit increase in the variable measuring exposure to this type of mistreatment. Further, perceptions of competitiveness (PERCOMP) at the educational institution were observed to negatively impact the mathematics outcomes achieved by students, whilst perceptions of cooperation (PERCOOP) had the opposite effect.

In the social ambit, the level of connection and attachment students reported to have with their parents was positively related with their Mathematics scores. This is evidenced by the coefficient associated with the SOCONPAR index, whilst the emotional support students perceived to receive from their parents (PAREMOSUP) did not have a meaningful impact on their Mathematics literacy.

Finally, variance values produced by the multilevel regression model pertaining to differences between educational institutions and variance due to differences between students within the same educational institution were 764.67 and 5884.69, respectively, with the corresponding deviance value being 187919.62. This suggests that, in comparison with the null multilevel regression model, the inclusion of dependent variables measuring student psychoemotional well-being significantly improved model fit and, as a result, its predictive power.

## 4. Discussion

Existing scientific literature strongly suggests that the psychoemotional state of individuals has an impact on the different ambits of their daily life. In the case of students, the study of the way in which psychoemotional well-being affects their academic life is of particular interest. In this context, the present work focused on identifying the variables capable of indicating psychoemotional health in Spanish students and the influence of these variables on performance in the subject of Mathematics through an examination of data reported in PISA 2018.

The present work highlights that students’ emotions are intimately related with their Mathematics performance. Effectively, emotional state, corresponding to the individual component of psychoemotional well-being in students, the academic or school component and the social component were confirmed to be related with the score or grade obtained in Mathematics. Present findings coincide with those presented by Elizondo-Moreno et al. (2018), in the sense that positive emotions were associated with higher scores in Mathematics and vice versa.

Within the group of predictors of the personal component of psychoemotional well-being examined in the present study, resilience, motivation towards achieving optimal learning and a sense of purpose in life stood out for the size of the coefficient associated with corresponding variables. The positive relationship between resilience and mathematics achievement reported in our paper was also reported by Gallesi and Matalinares Calvet (2012) and Donolato et al. (2020) in Peruvian and Italian students, respectively. As a consequence of this relationship, it would be useful for teachers to promote this trait in classrooms with the aim of achievement better Mathematics outcomes for their students. Although Mathematics teachers may suffer from a lack of training to achieve this outcome, the promotion of resilience in the classroom can start by, for example, supporting students to manage the frustration derived from tackling challenging subject material or motivating students through the appropriate methodologies, resources and activities. Motivating students in this way may also be beneficial if it serves to instill in students the desire to learn, which has been shown to have positive repercussions on Mathematics outcomes. With regards to the direction of the association between student Mathematics scores and the perception of having a clear objective or purpose in life, it is a potentially surprising finding that a negative association emerged when the opposite relationship may have been expected. As stated by Cohen and Cairns (2012), a sense of meaning in life is a hugely important element of individual well-being. Nonetheless, the process of searching to define this goal could become stressful and, therefore, translate into impinged well-being at the level of the individual, with this possibly having implications on the different contexts of their life, including the academic context. Another potential explication of this inverse association is related with the role played by Mathematics regarding student life goals. When Mathematics does not form part of the student’s life goals, neither as an outcome nor as a means, it makes sense that the relationship between Mathematics and goals or purpose in life would become counterproductive. However, work such as that of Erdem and Kaya (2021), also based on the analysis of PISA data, supports the results of our study by also reporting a negative effect, albeit of a smaller magnitude, between having a clear purpose in life and the mathematical literacy of Turkish students. In another sense, it also serves to highlight that a negative association was found between students’ perceptions of their appearance and Mathematics performance in the present study. Previously conducted scientific literature has barely examined the existing relationship between perception of physical appearance and Mathematics grades. In a broader sense and in consideration of the fact that satisfaction with external appearance is one of the main variables related with personal self-esteem (Broc, 2000), it can be confirmed that present findings contradict the majority of those reported by reviewed work conducted on the topic. Indeed, these studies evidence either a positive association or no association between self-esteem and performance in Mathematics (Bodkin-Andrews et al., 2010; Ros-Morente et al., 2017; Cid-Sillero et al., 2020).

With regards to the variables corresponding to the school and social component of the psychoemotional well-being of students for which significant outcomes were produced in the model predicting Mathematics scores, students’ parents played a key role. Such was this the case that a consolidated and positive link between parents and children counteracts, to some extent, the effect that negative experiences, such as those related with bullying at the academic institution, may have on the mathematics performance of students. In this sense, our findings are in line with those reported by Antonelli-Ponti et al. (2021) who pointed out a positive relationship between parental support and students’ mathematical literacy and with those highlighted by Rusteholz and Mediavilla (2022) in pointing out that bullying harms mathematical achievement. In addition, school climate, as measured in the present work in accordance with student perceptions of competitiveness and cooperation, was demonstrated to have a meaningful impact on the outcomes obtained by students in Mathematics, as proved by Gil-Madrona et al. (2019) in the area of science.

With regards to limitations of the present work, on the one hand, a number are inherent to the nature of the study design itself of the PISA. For instance, it was not mandatory to respond to questions regarding the psychoemotional state of students, making it impossible to examine the effect of other student traits, such as empathy or adaptability, on their performance in Mathematics. On the other hand, the significance of the variance between students and between schools indicates the existence of other sources of variability that were not controlled for in the present work and that may have an impact on student Mathematics performance. The magnitude of variability due to student differences was far greater than that found to exist between academic institutions and indicates that important variables were not examined that mainly pertain to student characteristics. Effectively, it is possible that, in addition to their psychoemotional well-being, other characteristics of the students’ personal and family context may also have a significant influence on the results they obtain in Mathematics. These variables were not included as predictors of the multilevel regression model developed in the present study as they were outside of the scope of the present work. Indeed, authors such as Molina-Muñoz et al. (2022) and Gamazo et al. (2018) have demonstrated that variables such as socioeconomic status and having to retake courses have an influence of the outcomes obtained by students in Mathematics. It is also even possible that the individual, social and family context to which students belong dictates the impact that variables of a psychoemotional nature have on Mathematics performance.

In conclusion, it is important to highlight the importance of psychological and emotional well-being in students and the need to support their emotional intelligence, alongside traditional cognitive intelligence, as both are likely to have a similarly meaningful impact on the academic outcomes achieved by students.

## Data availability statement

Publicly available datasets were analyzed in this study. This data can be found here: https://www.oecd.org/pisa/data/.

## Author contributions

DM-M, JC-G, and EM-P contributed to conception and design of the study. DM-M and JC-G organized the database. DM-M and EM-P performed the statistical analysis. DM-M wrote the first draft of the manuscript. JC-G and EM-P wrote sections of the manuscript. All authors contributed to the article and approved the submitted version.

## Funding

This research was partially funded by the research group “Datos, Educación y Sociedad” (PAIDI SEJ-622).

## Conflict of interest

The authors declare that the research was conducted in the absence of any commercial or financial relationships that could be construed as a potential conflict of interest.

## Publisher’s note

All claims expressed in this article are solely those of the authors and do not necessarily represent those of their affiliated organizations, or those of the publisher, the editors and the reviewers. Any product that may be evaluated in this article, or claim that may be made by its manufacturer, is not guaranteed or endorsed by the publisher.
